# The safety and feasibility of the screening for retinopathy of prematurity assisted by telemedicine network during COVID-19 pandemic in Wuhan, China

**DOI:** 10.1186/s12886-021-02018-x

**Published:** 2021-06-11

**Authors:** Zheng Guo, Nan Ma, Yixuan Wu, Hua Yuan, Wanjun Luo, Lingkong Zeng, Hong Jie, Shilian Li

**Affiliations:** 1 Department of Ophthalmology, Wuhan Children’s Hospital (Wuhan Maternal and Child Healthcare Hospital), Medical College, Huazhong University of Science & Technology, Wuhan, 430016 China; 2 Department of infection management, Wuhan Children’s Hospital (Wuhan Maternal and Child Healthcare Hospital), Tongji Medical College, Huazhong University of Science & Technology, Wuhan, 430016 China; 3 Department of neonatology, Wuhan Children’s Hospital (Wuhan Maternal and Child Healthcare Hospital), Tongji Medical College, Huazhong University of Science & Technology, Wuhan, 430016 China

**Keywords:** Retinopathy of prematurity, Screening, COVID-19, Telemedicine

## Abstract

**Background:**

During the coronavirus disease 2019 (COVID-19) epidemic, due to the traffic blockade and the shortage of medical resources, more and more premature infants could not receive timely and effective ROP screening, which delayed treatment and even caused children blindness. Therefore, how to carry out ROP screening safely and effectively during the epidemic was very important and urgent. This study aimed to evaluate the safety and feasibility of ROP screening assisted by telemedicine network during COVID-19 outbreak.

**Methods:**

This retrospective study was conducted at Wuhan Children’s hospital in Wuhan, China, from January to October, 2020. The measures which were performed to make the ROP screening more safe and effective were summarized and the comparison between ROP screening assisted by telemedicine network in 2020 and usual screening in 2019 were analyzed.

**Results:**

A total of 267 outpatient infants completed ROP screening. The median gestational age was 32 weeks (30w to 34w) and the median birth weight was 1780 g (1460 g to 2100 g). Meanwhile, 149 (55.8%) out of 267 infants were males. During January to May in 2020, 86 screening appointments were received, among which 67 (77.9%) were from telemedicine platform online. The completing percentage of total online ROP appointments was higher than that of total face-to-face appointments (58.1% VS 22. 1%, *P* = 0.018). As for the number of infants screened between 2020 and 2019 from Februaryto October, 54 infants completed ROP screening in 2020, which was higher than that (51participants) in 2019 on September. Furthermore, compared with the usual screening in 2019, ROP screening assisted by telemedicine network in 2020 had smaller gestational age (32w VS 33w, *p<0.001*) and lower birth weight (1780 g VS 1900 g, *p = 0.001*). However, of the 267 infants screened, 18(6.7%) had ROP while the percentage of ROP screened in 2019 was the same (44[6.7%]). During follow-up, none of medical staffs was infected and no adverse reaction was reported.

**Conclusions:**

The screening for retinopathy of prematurity assisted by telemedicine network was safe and feasible during the COVID-19 pandemic. Preventive measures before and after screening were very necessary, which could effectively avoid cross infection.

Retinopathy of prematurity (ROP) is one of the severe diseases of childhood blindness, accounting for 6–18% worldwide [1].ROP screening for premature infants as soon as possible is necessary and significant. During the coronavirus disease 2019 (COVID-19) pandemic, ROP screening was presented with various challenges, such as how to prevent cross infection, how to ensure the accuracy and timely of the examination and how to better communicate with the legal guardians of infants. Telemedicine ROP screening provided a possibility that appointments, diagnosis, treatment suggestion and follow-up could be completed online. It had been reported previously [2], but had not been performed during the epidemic. Therefore, in this study, we aimed to evaluate the safety and efficacy of ROP screening assisted by telemedicine network during the COVID-19 outbreak and to provide a reference for ROP screening if a similar public health emergency occurs.

## Subjects and methods

### Subjects

A total of 267 infants completed ROP screening from outpatient clinic during COVID-19 pandemic in Wuhan Children’s Hospital from January to October, 2020. The median birth weight was 1780 g (1460 g to 2100 g) with median gestational age 32 weeks (30 w to 34 w). Of the 267 participants, 149 (55.8%) were males. According to guidelines for screening of retinopathy of prematurity in China (2014) [3], ROP screening criteria included low gestational age and low birth-weight (< 2000 g or gestational age < 32 weeks). Meanwhile, screening range was extended by pediatric ophthalmologists for the premature infants with severe systematic disease or longtime oxygen inhalation. The follow-up continued until peripheral retina angiogenesis. A written informed consent was obtained from parents or the other legal of guardians before screening. This study was approved by Ethics Committees of Wuhan Children’s Hospital.

## Methods

### Epidemiological data of COVID-19 before ROP screening

During COVID-19 pandemic, epidemiological information about the infants and their family members were collected: 1.The respiratory symptoms in 14 days, such as fever, cough, fatigue, or diarrhea and so on. 2. The ocular symptoms in 14 days, such as conjunctival congestion, conjunctival discharge, eyelid swelling and so on. 3. Contact history in 14 days with suspicious or diagnosed COVID-19 patients. Meanwhile, the test of severe acute respiratory syndrome coronavirus 2 (SARS-CoV-2) real-time reverse transcriptase–polymerase chain reaction (RT-PCR) of nasopharynx or oropharynx swabbing specimen were recommended for all infants before ROP screening. Infants with positive results would be hospitalized at isolation ward and get a bedside screening if needed. The infants without COVID-19 had a successful appointment for ROP screening out of hospitalization.

**Fig. 1:**
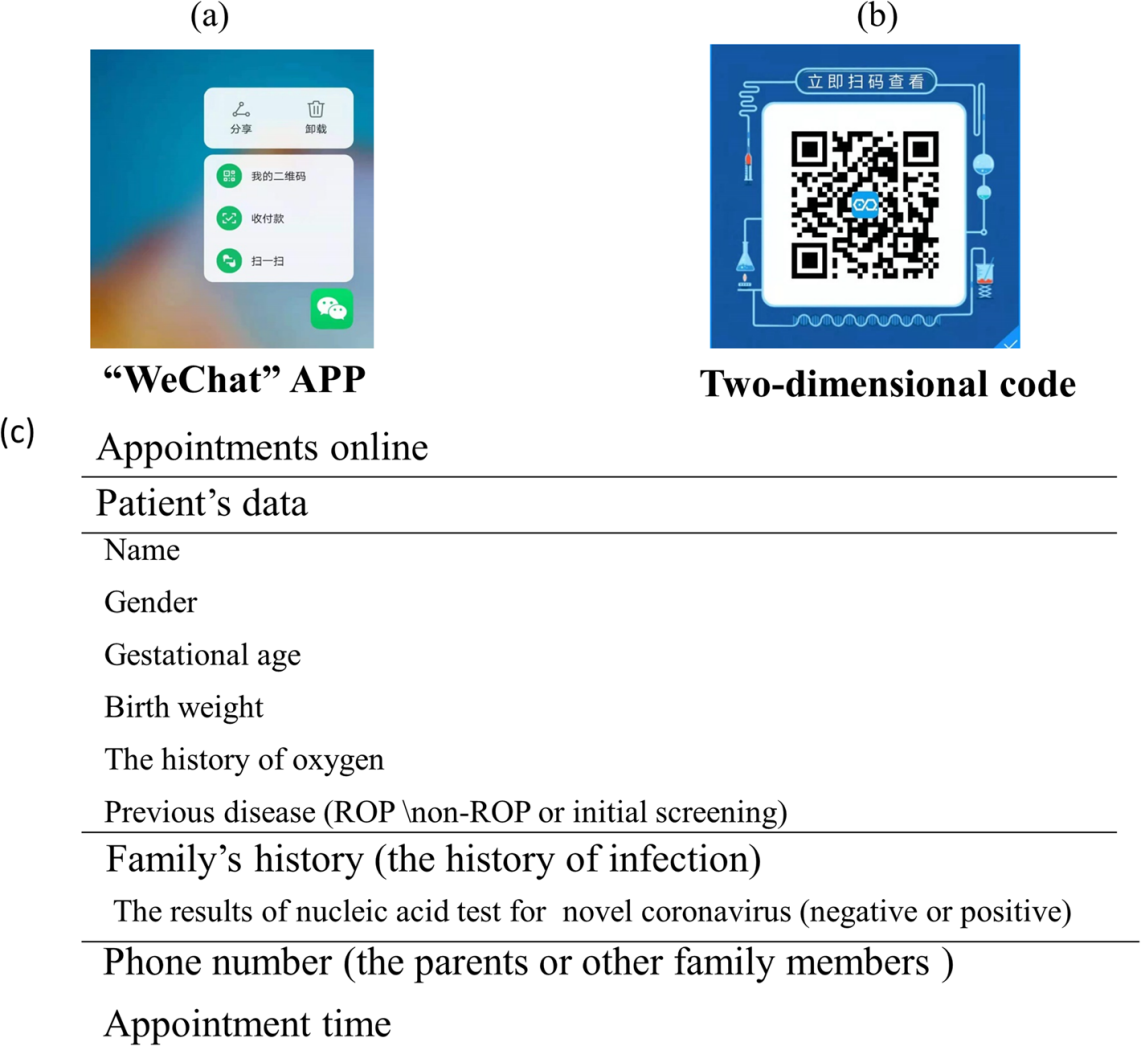
The WeChat Software Interface for Making Appointments of Screening for Retinopathy of Prematurity. 1a.The WeChat app was designed for smartphone or i-pad. Parents could conduct online consultation through entering into the official website of hospital WeChat public account. 1b.Parents could scan the two-dimensional code to make appointments online.1c.The information which parents need complete in the reservation interface when making an online appointment

### Establishment of telemedicine ROP screening platform

A mobile online consultation group was set up for ROP screening which contained pediatric ophthalmologists, ophthalmic specialist nurses, neonatal pediatricians, professors from other hospitals and experts from emergency or infection department. Meanwhile, a two-dimensional code created by WeChat software was available. Once registered the general information for e-health in the official website of hospital, parents could schedule ROP screening time through scanning the two-dimensional code at home or anywhere (as Fig. 1). Furthermore, an online telemedicine platform was created by network information department of Wuhan children’s hospital. Parents could be able to upload text, audio, video, images and even conduct a conversation with pediatric ophthalmologists via telemedicine platform after entering into the official website of hospital WeChat public account. After that, an ophthalmic specialist nurse checked and updated screening information, contacted the family members, informed them of the exact screening time, procedures and precautions for examination. Moreover, during follow-up time, the parents could keep in touch with pediatric ophthalmologists and get professional guidance through the online clinic and telemedicine consultation. As the compliance of online appointment among parents of premature infants was increasing, all the screening appointments were received from telemedicine platform since June.

### The process of ROP screening

After entering the examination room, the legal guardian of infants should provide negative results of both adults and pediatric patients of COVID-19 nucleic acid test, blood analysis and C-reactive protein within 2 week. Then the legal guardian of infants signed informed consent for ROP screening. Before and after screening, body temperatures were recorded for the infants and adults by specialist nurse. Care pads were disposable and replaced at the end of each screening. Pediatric ophthalmologists adopted III protection measures during screening through a wide field fundus digital camera system (RetCam III Clarity Medical Systems, USA). Furthermore, an air disinfection machine (KDSJ-Y1200, Chengdu Laoken Scientific and Technology Co. Ltd.) kept running to disinfect air in the examination room. Immediately after each exam, all surfaces especially the instrument and equipment were wiped-down at least twice lasting 1 min with 75% alcohol for disinfection purpose. The lens of the equipment was cleaned with sterile cotton balls to remove coupling agents. The tips of the lens were wiped down with cotton balls soaked in 75% alcohol. Cleaning was performed three times, each time lasting at least 1 min. At the end of each day, the floor and all other surfaces were wiped down with 1000 mg/L chloride solution for at least 30 min. After that, ultraviolet (uv) lighted for at least 60 min with doors and windows closed to disinfect the air. At last, open windows to air the room.

### Statistical analysis

All statistic data were analyzed with SPSS software, version 25.0. The continuous variables were represented by means and standard deviation if the data were normally distributed. Otherwise, the continuous variables were represented by median and percentiles which were compared by Mann-Whitney U test. Categorical data were expressed as percentages and counts which were compared by χ^2^ test or Fisher’s exact test. A *P* < .05 (2-sided) was considered statistically significant.

## Results

### Comparison the completing rate between online and face-to-face appointments for actual ROP screening during the early stage of the COVID-19 epidemic

The data of ROP appointments and screenings between online and face-to-face consultation from January to may in 2020 were analyzed in Table 1. A total of 86 appointments were received, of which 69(80.2%)had completed ROP screening from January to may, 2020. Meanwhile, of the 86 appointments, 67(77.9%)were from telemedicine scheduling via online and 19(22.1%)from outpatient clinic by face to face. As shown in Table 1, the completing percentage of total online appointments was higher in compared with that of total face-to-face appointments (58.1% VS 22.1%, *P* = 0.018). However, there were no significant differences in the monthly data between online and face-to-face screening

**Table 1:** Online and Face-to-face Appointments for ROP Screening from January to May in 2020

Month	Total				Online		Face-to-face		*P* Value
	screened (No.)	appointment (No.)	Online (No. %)	Face-to-face (No. %)	Screened (No.)	Appointment (No.)	Screened (No.)	Appointments (No.)	
Jan.	0	0	0	0	0	0	0	0	–
Feb.	0	18	0	0	0	18	0	0	–
Mar.	25	18^a^ + 21	24 (61.5)	1 (2.6)	24	18^a^ + 20	1	1	> 0.99^b^
Apr.	22	23	14 (60.9)	8 (34.8)	14	15	8	8	> 0.99^b^
May.	22	24	12 (50.0)	10 (41.7)	12	14	10	10	0.493^c^
Total	69	86	50 (58.1)	19 (22.1)	50	67	19	19	0.018^c^

ROP: Retinopathy of Prematurity; Jan: January; Feb: February; Mar: March; Apr: April; May: May

a In February, 18 appointments were received but none of them completed screening

b Fisher’s exact probability test

c: χ2 Test

### Comparison the number of premature infants of screening for retinopathy of prematurity between 2019 and 2020 from February to October

**Fig. 2:**
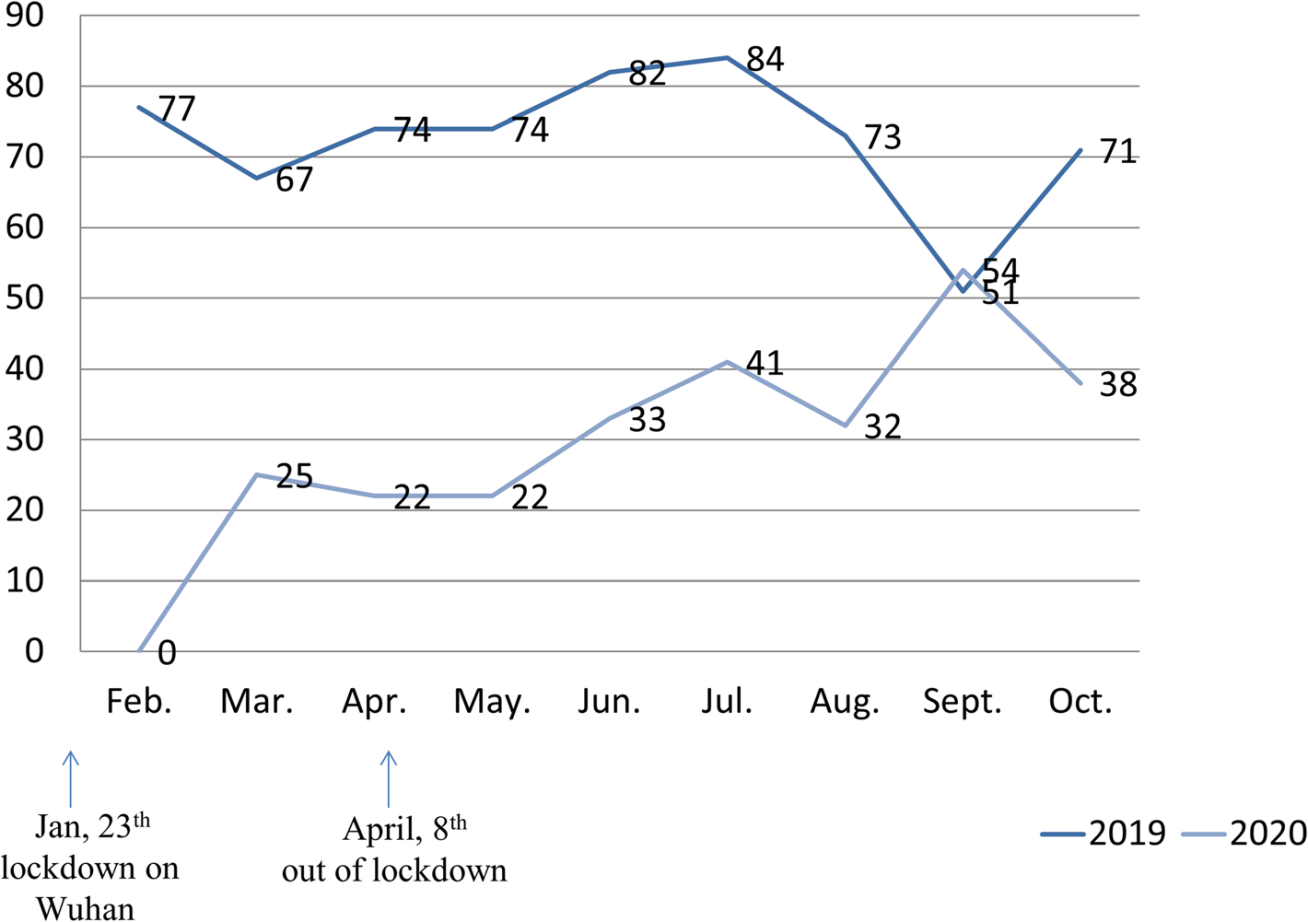
Comparison the Number of Infants of Screening for Retinopathy of Prematurity between 2019 and 2020 from February to October. Jan: January; Feb: February; Mar: March; Apr: April; May: May; Jun: June; Jul: July; Aug: August; Sept: September; Oct: October

The data of ROP screening in 2019 and 2020 were summarized in Fig. 2. As Wuhan city was lockdown on January 23th in 2020, the number of infants screened was significantly lower than those in 2019. Fortunately, with the development of telemedicine and online consultation, the percentage of infants screened was increasing gradually. In September, 54 infants completed ROP screening in 2020, which was higher than that (51 participants) in 2019.

### Comparison the demographic and clinical data of premature infants of screening for retinopathy of prematurity between usual screening procedure in 2019 and screening assisted by telemedicine network in 2020

The demographic characteristics and clinical manifestations of ROP screening from February to October in 2019 and 2020 were shown in Table 2. The gestational age of infants screened in 2020 (median age 32 weeks) was smaller than that in 2019 (median age 33 weeks) (P<0.001). Meanwhile, the birth weight of infants screened in 2020 (median weight 1780 g) was lower than that in 2019 (median weight 1900 g) (*P* = 0.001). Especially, of 267 infants in 2020, 92 pediatric patients (34.5%) had birth weight which was higher than 2000 g, while among 653 infants in 2019, 303 pediatric patients (46.4%) had the higher birth weight. The difference between the 2 incidences had statistical significance (P = 0.001). As for the ocular findings, the percentage of ROP screening in 2020 was 9.4% (25 participants), including ROP (18[6.7%]), cytomegalovirus retinitis (3[1.1%]), retina hemorrhage (3[1.1%]) and optic disc dysplasia (1[0.4%]). While of the 653 infants screened in 2019, 48(7.4%) had ocular disorders, which contained ROP (44[6.7%]), cytomegalovirus retinitis (1[0.2%]), retina hemorrhage (3[0.5%]). However, no difference was found for the outcomes of screening between the 2 groups in 2019 and 2020. Furthermore, 154 pediatric patients (57.7%) in 2020 were from other cities. Unfortunately, the data of traffic for infants screened in 2019 was unrecorded.

**Table 2:** Demographic and Clinical Characteristics of Infants for Retinopathy of Prematurity Screening between 2019 and 2020 from February to October

Demographic and clinical characteristics	Screening assisted by telemedicine network in 2020 No. (%)(*N* = 267)	Usual screening in 2019 No. (%)(*N* = 653)	P value
Male	149 (55.8)	375 (57.4)	0.652^c^
Gestational age, median (IQR), weeks	32 (30 to 34)	33 (31 to 34)	***<0.001***^***d***^
Birth weight, median (IQR),g	1780 (1460 to 2100)	1900 (1510 to 2300)	***0.001***^***d***^
Birth weight group, g			***0.003***^***c***^
<1500	72 (27.0)	136 (20.8)	0.043^c^
1500 to <2000	103 (38.6)	214 (32.8)	0.093^c^
≥ 2000	92 (34.5)	303 (46.4)	***0.001***^***c***^
Ocular findings			
Total	25 (9.4)	48 (7.4)	0.305^c^
Zone III Phase 1	12 (4.5)	35 (5.4)	0.588^c^
Zone III Phase 2	3 (1.1)	1 (0.2)	0.076^b^
Zone II Phase 1	1 (0.4)	1 (0.2)	0.496^b^
Zone II Phase 2	1 (0.4)	5 (0.8)	0.678^b^
Zone II Phase 2 Plus	1 (0.4)	2 (0.3)	>0.99^b^
CMV retinitis	3 (1.1)	1 (0.2)	0.076^b^
Retina hemorrhage	3 (1.1)	3 (0.5)	0.364^b^
Optic disc dysplasia	1 (0.4)	–	–
Traffic (come from other cities)	154 (57.7)	NA	–

ROP: Retinopathy of Prematurity; CMV: Cytomegalovirus; NA: not available

b Fisher’s exact probability test

c: χ^2^ Test

d: Mann-Whitney U test

### Follow-up

Follow-up time was 3 days, 2 weeks and 4 weeks after screening. During follow-up, no COVID-19 infection, conjunctivitis, or other adverse reactions were reported. Furthermore, all health workers and medical staffs had negative results of novel coronavirus nucleic acid test and serum antibodies test. Seventeen out of 267 had suspended screening by themselves. Among the 17 cases, 9(52.9%) complained that their house was too far away from Wuhan and the traffic was not convenient enough during COVID-19 pandemic. The remaining reasons included rejecting nucleic acid tests (1[5.9%]), screening in other hospitals (4[23.5%]) and personal reasons (3[17.7%]).

## Discussion

As the COVID-19 pandemic continued to spread around the world, it had proven difficult to continue screening for ROP safely and efficiently. At the early stage of COVID-19 pandemic in China, especially the period of lockdown time on Wuhan, the National Healthcare Committee encouraged medical institutions to take advantage of telemedicine and standardize online medical consultation services in order to reduce cross-infection and avoid patients gathering. As we known, through telemedicine, fundus imaging could be conducted by non-ophthalmologists and then transmitted to ROP experts for screening [2, 4–8]. Furthermore, telemedicine system also allowed us to offer medical services when outpatient activities were severely restricted and reduced the burden on hospital staff, such as completed risk assessment, scheduled surgery and conducted follow-up [4, 6, 9]. Many previous reports had demonstrated the importance and advantages of telemedicine in ophthalmology since COVID-19 outbreak [10, 11]. However, most of the reports evaluated outcomes of telemedicine for adults [8, 10, 12], instead of children. Furthermore, little is known about the safety and feasibility of ROP screening assisted by telemedicine network for pediatric patients during COVID-19 pandemic.

As the retinopathy of prematurity could lead to childhood blindness, how to make appointments of screening efficiently was very importance. In our study, parents could make appointments of ROP screening through telemedicine platform at home or any other place instead of ophthalmologist’s clinic in hospital, which could decrease the travelling expenses for the first outpatient consultation. Meanwhile, compared with usual appointments, appointments on line could decrease the number of direct contacts between patients and physicians which could avoid potential risk of cross-infection. In our study, among the total appointments, the actual completing percentage through online was higher than that via face-to-face during January to May in 2020 (58.1% VS 22.1%, *P* = 0.018), which demonstrated the advantage of convenience and efficiency of appointments for ROP screening assisted by telemedicine platform. Meanwhile, with the widespread application of telemedicine, the monthly number of infants screened was increasing. In our study, it was statistically analyzed that the data of infants for ROP screening on September in 2020 was higher than that in 2019. One hypothesis was that young parents preferred to accept telemedicine platform and online appointments which was more safe and convenient. Another reason was perhaps that with climate warming and epidemic ease, personal fear about novel coronavirus relieved.

The accuracy of ROP screening was another challenge during COVID-19 pandemic. In our study, the ophthalmologist who performed the ROP screening was also a specialist of pediatric fundus disease during COVID-19 pandemic, which could shorten diagnosis and referral time for ROP in order to prevent missing severe ROP cases and optimal treatment time. Moreover, according to the information provided by parents on telemedicine platform, the assessment for potential retinopathy of premature infants were performed by the pediatric ophthalmologist and the infants with lower weight, lower gestational age or poorer general condition was given priority to be checked. Maybe that was the reason why the infants screened in 2020 had smaller gestational age and lower birth weight than that in 2019. Parents could also upload photos, texts even videos on telemedicine platform, which could enrich the information of online consultation and improved the diagnosis rate. Compared with usual screening, the ocular outcomes of ROP screening in 2020 and 2019 had no statistic difference, which further showed the feasibility of ROP screening assisted by telemedicine network.

In our study, novel coronavirus nucleic acid test was essential for all the infants before ROP screening but serological tests of novel coronavirus were not demanded. The main reason was that serological indicators ordinarily reflected previous infection instead of current infection. Meanwhile, it was safer to evaluate epidemiological history and travel history of caregivers of infants, especially within 14 days. Infants with a suspected history of contact were demanded to collect not only nasopharyngeal swabs but also tear swabs for novel coronavirus nucleic acid testing. Furthermore, there was a special clinic with air disinfection for ROP screening, which was away from internal medical clinic especially the fever clinic. Whether to take chest computed tomography (CT) before ROP screening was still controversial. It was reported that the lung CT of COVID-19 were usually atypical in neonates [13]. The radiation of CT test was another reason. Thus none of the infants took chest CT before screening in our study. Fortunately, none of medical staff infected novel coronavirus 2019 though fathers of 2 infants had COVID-19 confirmation. The information was collected through online consultation that the 2 infants had never contact with their COVID-19 father since they were born, which demonstrated the necessary of epidemiological investigation before ROP screening.

In our study, the incidence of ROP screening both in 2019 and 2020 was 6.7%, lower than previous reports [1, 14], which may be due to the reason that the infants without systematic diseases in outpatient clinic had relatively high gestational ages and birth weights. It was well-known that low gestational age and birth weight were still the major risk factors for ROP [15, 16]. In North America, new ROP screening criteria [17, 18] include gestational age < 28 weeks and weight gain at 40 days after birth, which had higher sensitivity and specificity for ROP type 1 than current criteria, and lead to 30% less screening population [17]. However, in our study, we adopted the guidelines for screening of retinopathy of prematurity in China (2014, 3],which stipulated ROP screening standard of birth weight less than 2000 g or gestational age younger than 32 weeks.

There were many limitations in our study. First, no satisfaction survey was conducted on the legal of guardians of infants participating in telemedicine screening. Second, the follow-up time was short which lacked the long-term outcomes. Third, infants were not demanded to take lung CT or COVID-19 serum antibodies tests before screening, which increased the risk of infection for medical staffs. Therefore, it was recommended that medical staffs would take novel coronavirus nucleic acid testing every two weeks. Nevertheless, this study was clinical significant for ROP screening, which provided evidence for effects and safety of ROP screening assisted by telemedicine network during COVID-19 outbreak.

## Conclusion

Screening for retinopathy of prematurity assisted by telemedicine network during the COVID-19 pandemic is safe, effective and feasible. Through detailed online consultation of epidemiological history and general information of infants, pediatric ophthalmologist can make assessment and give priority to screen the infants with smaller gestational age, lower weight or poorer general condition. Furthermore, appropriate prevention and control measures can effectively prevent cross-infection for medical staffs during screening.

## Data Availability

The datasets generated during and/or analyzed during the current study are not publicly available due to participants identity revealing data but are available from the corresponding author on reasonable request.

## Abbreviations

ROP: Retinopathy of Prematurity
COVID-19: coronavirus disease 2019
SARS-CoV-2: severe acute respiratory syndrome coronavirus 2
CT: computed tomography
RT-PCR: real-time reverse transcriptase–polymerase chain reaction;
Jan: January
Feb: February
Mar: March
Apr: April
May: May
CMV: Cytomegalovirus
Jun: June
Jul: July
Aug: August
Sept: September
Oct: October
